# Impact of depressive disorders on quality of life after middle ear surgery in patients with chronic otitis media

**DOI:** 10.1007/s00405-020-06397-7

**Published:** 2020-10-03

**Authors:** Susen Lailach, Theresa Langanke, Thomas Zahnert, Susan Garthus-Niegel, Marcus Neudert

**Affiliations:** 1 Faculty of Medicine Carl Gustav Carus, Department of Otorhinolaryngology, Head and Neck Surgery, Technische Universität Dresden, Fetscherstraße 74, 01307 Dresden Saxony, Germany; 2 Department of Medicine, Faculty of Human Sciences, Medical School Hamburg, Hamburg, Germany; 3 Faculty of Medicine Carl Gustav Carus, Institute and Policlinic of Occupational and Social Medicine, Technische Universität Dresden, Fetscherstraße 74, 01307 Dresden, Saxony, Germany; 4 Department of Child Health and Development, Norwegian Institute of Public Health, Oslo, Norway

**Keywords:** Depressiveness, Tympanoplasty, Cholesteatoma, Quality assessment, Middle ear reconstruction

## Abstract

**Purpose:**

The aim of this study was to determine whether preoperative depressive symptoms influence health-related quality of life (HRQOL) after middle ear surgery in patients with chronic otitis media (COM).

**Methods:**

This prospective clinical case study was conducted at a tertiary referral center. All 102 patients who had undergone middle ear surgery for COM were assessed clinically and by audiometric testing (pure tone audiometry) in pre- and postoperative settings. Disease-specific HRQOL was assessed by the validated chronic otitis media outcome test 15 (COMOT-15) and the Zurich chronic middle ear inventory (ZCMEI-21). General HRQOL was measured using the short form 36 (SF-36). Depressive symptoms were assessed using the patient health questionnaire (PHQ-D). The Charlson comorbidity index (CCI) was used to classify comorbidities. The middle ear status was determined using the ossiculoplasty outcome parameter staging (OOPS) index.

**Results:**

After middle ear surgery, the total COMOT-15 and ZCMEI-21 scores improved significantly (*p* < 0.001). General HRQOL (total SF-36 score) was unaffected by surgery (*p* < 0.05). Patients without elevated depressive symptoms had significantly better total scores for the COMOT-15 (*p* < 0.01), ZCMEI-21 (*p* < 0.001), and for SF-36 (*p* < 0.001) postoperatively. The results of the multiple regression analyses show that, after adjusting for the OOPS, CCI, and hearing improvement, preoperative depressiveness was significantly associated with worse postoperative COMOT-15 and ZCMEI-21 outcome scores (*β* = 0.425 and *β* = 0.362, *p* < 0.001).

**Conclusion:**

Preoperative depressiveness was an essential predictive factor for HRQOL in patients with COM. This should be considered during patient selection to provide more suitable preoperative counseling.

## Introduction

Chronic otitis media (COM) is a common disease that affects 0.45–2.6% of the population [1, 2]. Patients with COM suffer from ear discharge, pain, and hearing impairment; due to extensive communication problems, this results in social and emotional restrictions. Additionally, COM leads to restrictions in daily activities and social interactions and increased use of the healthcare system [3–6].

For several years now, health-related quality of life (HRQOL) measurements have been an increasingly important outcome parameter even after middle ear surgery [7]. Previous studies mainly focused on the development and validation of measurement instruments as well as the evaluation of pathology-associated or surgery-associated influencing factors [3, 5, 8–11]. Especially in patients who have a reduced disease-specific HRQOL despite good hearing and normal ear findings, the extent to which patient-related factors modulate disease-specific HRQOL remains unclear.

Depression has long been associated with hearing disorders [12–14]. However, the coexistence of mood disorders in patients with COM was analyzed in only a few studies [15, 16]. Particularly, studies in orthopedics and pain therapy demonstrated that patients with mental health disorders have been associated with less postoperative improvement [17, 18]. In patient-reported outcome measures (PROMs), patients with anxiety and depression felt that interventions were less beneficial compared to others without these comorbidities [19–22]. Given that depressiveness affects the perception of chronical illness [23–25], depressive disorders may modulate the relationship between COM symptom burden and the decrease in HRQOL in patients with COM.

As healthcare continues its transition to evidence-based medicine, surgeons must identify and analyze individual patient-related factors that affect treatment outcomes. By understanding how patient characteristics affect surgical outcomes, surgeons can achieve more prudent patient selection while providing better preoperative advice.

The aim of this study is to determine whether preoperative depressive symptoms influence PROMs after middle ear surgery for patients with COM.

## Patients and methods

### Study design and population

Between July 2017 and December 2018, 102 patients with COM (with or without cholesteatoma) were prospectively enrolled. They underwent primary or revision surgery at a tertiary referral center. Patients with and without postoperative complications were included. Preoperative assessment was performed 1 day before surgery. Treatment evaluation was performed 6 months postoperatively. Patients who missed the control visit after 6 months were excluded. Pre- and postoperative evaluation included HRQOL measurement, screening for the presence and severity of depression, registration of comorbidities, and pure tone audiometry.

### Surgical technique

In all patients, a retroauricular incision was used. Tympanic membrane reconstruction was performed via an underlay technique using fascia and cartilage slices. In patients with cholesteatoma, a sequential surgical strategy was preferred, as described previously [11]. All open mastoid cavities were obliterated with autologous bone pate or bone replacement material (bioactive glass S53P4) and sliced concha cartilage plates. The ossicular chain was reconstructed using titanium partial ossicular replacement prostheses (PORP: titanium clip prosthesis, titanium ankle prosthesis, titanium bell prosthesis) or total ossicular replacement prostheses (TORP: Aerial Type Düsseldorf, malleus notch TORP, all Kurz Company, Dusslingen, Germany). Hearing reconstruction and cholesteatoma removal were routinely performed in a single-stage procedure.

### HRQOL measurement

Disease-specific HRQOL measurement was performed using the validated and disease-specific chronic otitis media outcome test 15 (COMOT-15) and the Zurich chronic middle ear inventory (ZCMEI-21) in German language. The short form 36 (SF-36) was used to determine general HRQOL.

I. COMOT-15
  The COMOT-15 has been previously validated in German language and included 15 Likert scaled items [8]. The survey is subdivided into three subscores: “ear symptoms” (items 1–6), “hearing function” (items 7–9), and “mental health” (items 10–13). From this, an overall score was determined (items 1–13). Additionally, a general evaluation of disease-specific HRQOL (item 14) and the frequency of physician consultations (item 15) was conducted. As described previously, COMOT-15 scores were normalized to a scale from 0 to 100 by dividing the sum of the raw scores of the items by the sum of spans of the items followed by multiplying by 100 [8]. Higher scores indicated a poorer HRQOL.
II. ZCMEI-21
  The ZCMEI-21 has been previously validated in German language [9] and included four subscales: “ear signs and symptoms” (items 1–5), “hearing function” (items 6–10), “psychosocial impact” (items 11–18), and the “use of medical resources” (items 19–21). All answers are presented using a 5-point Likert scale. Higher scores indicated a poorer HRQOL.
III. SF-36
  The German version of the SF-36 was validated by Bullinger [26] The SF-36 records eight aspects of subjective health: “physical functioning” (10 items), “role-functioning physical” (4 items), “bodily pain” (2 items), “general health” (5 items), “vitality” (4 items), “social functioning” (2 items), “role-functioning emotional” (3 items), and “mental health” (5 items). Data were scored according to the SF‐36 Analysis and Interpretation Manual [27]. The scales were scored from 0 (lowest level of functioning) to 100 (highest level of functioning).

### Depression screening

The German version of the patient health questionnaire (PHQ-D) [28] was used to assess the severity of depressive symptoms. The PHQ-D has been developed to screen for mental disorders in primary care, using diagnostic criteria from the fourth edition of the American Psychiatric Association ‘s Diagnostic and Statistical Manual of Mental Disorders (DSM-IV). The depression module (PHQ-9) of the PHQ-D is a screening tool used in numerous patient populations to quantify the severity of patient-reported depressive symptoms. Patients rate the occurrence of depressive symptoms within the last two weeks on a 4-point scale from 0 (“not at all”) to 3 (“nearly every day”). The severity of depressive symptoms was analyzed as a continuous score from 0 (none) to 27 (severe). Additionally, the categorical algorithm was applied to distinguish between patients with and without depressive symptoms. For the categorical algorithm, the answers to the questions were dichotomized: “not at all” (0) and "several days“ (1) were coded as 0 (“symptom absent”), and the answers “more than half the days” (2) and “nearly every day” (3) were coded as 1 (“symptom present”). According to the PHQ-D manual [28], patients were classified as “depressed” when at least five symptoms were present and at least one symptom was “depressed feelings” or “loss of interest”.

### Comorbidity and disease-specific parameters

The Charlson comorbidity index (CCI, Table 1) comprises 17 comorbidities, which are weighted (from 1 to 6) based on the adjusted risk of mortality or resource use [29]. The sum of all the weights results in a single comorbidity score for each patient.

**Table 1:** Charlson comorbidity index (CCI)

Comorbid conditions	CCI weights
Myocardial infarction	0
Congestive heart failure	2
Peripheral vascular disease	0
Cerebrovascular disease	0
Dementia	2
Chronic pulmonary disease	1
Rheumatic disease	1
Peptic ulcer disease	0
Mild liver disease	2
Diabetes without chronic complications	0
Diabetes without chronic complications	1
Paraplegia or hemiplegia	2
Renal disease	1
Any malignancy without metastasis	2
Moderate or severe liver disease	4
Metastastic malignoma	6
AIDS /HIV	4

The ossiculoplasty outcome parameter staging (OOPS) index [30] was designed to provide a risk and outcome analysis in the context of the middle ear environment (Table 2). The calculation is based on intraoperative middle ear findings and preoperative examination. The OOPS index was used to evaluate the individual severity of the middle ear disease.

**Table 2:** The ossiculoplasty outcome staging (OOPS) index

Risk factor		Risk value
Drainage	None	0
	Present > 50% of the time	1
Mucosa	Normal	0
	Fibrotic	2
Ossicular chain	Normal	0
	Malleus +	1
	Malleus -	2
Type of surgery	No mastoidectomy	0
	Intact canal wall mastoideectomy	1
	Canal wall down mastoidectomy	2
Revision surgery	No	0
	Yes	2

### Audiological assessment

According to the Committee on Hearing and Equilibrium guidelines, pre- and postoperative air conduction (AC) thresholds were calculated as an average (pure tone average, PTA) over the frequencies 0.5, 1, 2, and 3 kHz [31].

### Statistical analyses

Descriptive statistics including mean and standard deviation (SD) were used to represent demographic and outcome data. Normality of the outcome scores was assessed using the Kolmogorov–Smirnov test. For normally distributed outcome scores, a paired *t* test was used to compare the mean scores pre- and postoperatively. Mean scores between two different groups were analyzed using an independent *t* test.

Multiple linear regression analyses were conducted to determine the effect of preoperative depressiveness on outcome scores, controlling for comorbidity (CCI), middle ear pathology (OOPS index), and improvement in the AC. A *p* value < 0.05 was considered statistically significant.

### Sample size and approval

The number of patients was based on a statistical sample size calculation (statistical power 0.8, alpha = 0.05, *n* = 98). Institutional review board approval was obtained before study initiation by the local ethical review committee (EK 166,042,017). All patients gave their informed consent. The study was conducted in accordance with the Declaration of Helsinki 1964.

## Results

This study included 102 patients (50 males and 52 females) with a mean age of 49.32 ± 16.04 years. Due to the high response rate (102/132 patients, 77%) and similar gender and age distribution, no response bias was present. Fifty-six (55%) patients underwent revision surgery, 62 patients (60.8%) presented with chronic mesotympanic otitis media, and 40 patients (39.2%) suffered from cholesteatoma. Eighteen (17.6%) patients underwent canal wall down surgery. In 84 (82.4%) patients, an intact canal wall technique was performed.

In total, 44 patients (43.1%) had an intact ossicular chain. Hearing reconstruction was performed by PORP in 23 (22.5%) patients and by TORP in 26 patients (25.5%). Due to middle ear pathology (fixed or destroyed stapes footplate), primary reconstruction of the ossicular chain was not performed in nine patients.

**Fig. 1:**
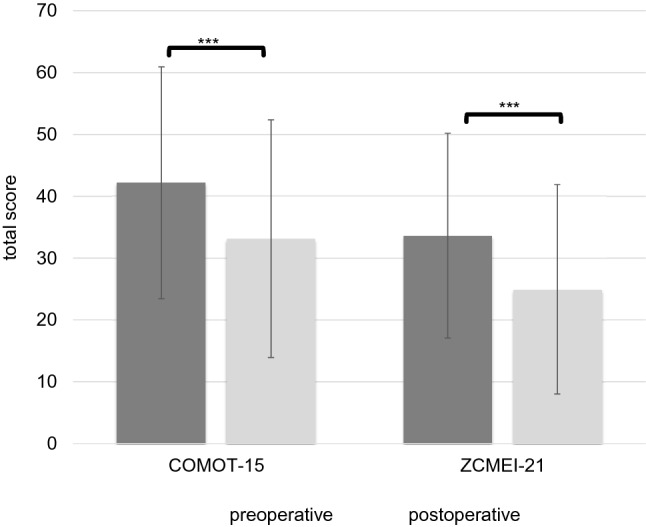
COMOT-15 and ZCMEI-21 total score: comparison of pre- and postoperative results (*n* = 102); Data shown are mean ± SD; ***p ≤ 0.001 preoperative vs. postoperative

**Fig. 2:**
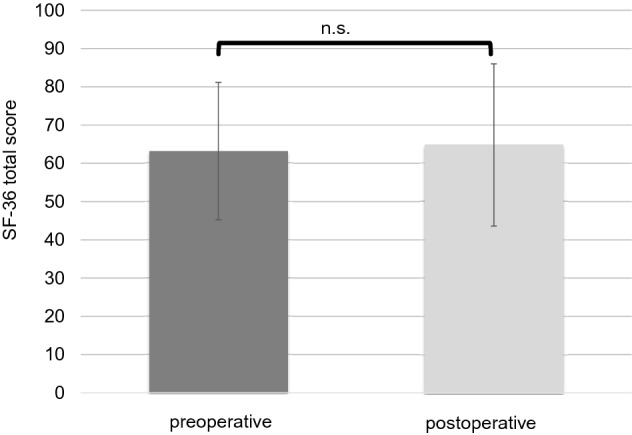
SF-36 total score: comparison of pre- and postoperative results (*n* = 102); Data shown are mean ± SD; *n.s.* not significant (*p* > 0.05)

**Fig. 3:**
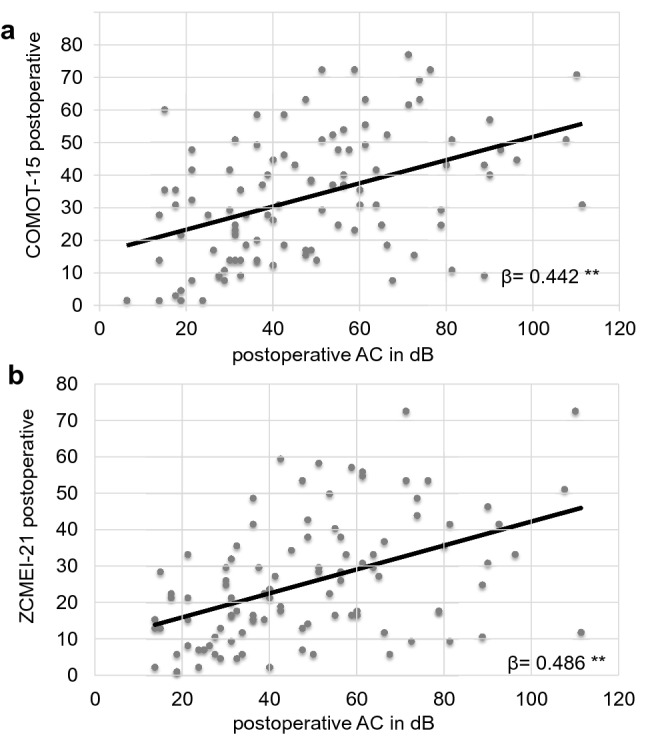
Postoperative assessment of COMOT-15 (**a**) and ZCMEI-21 (**b**): bivariate regression between total scores and postoperative air conduction threshold (AC); *β* regression coefficient, **p ≤ 0.01)

**Fig. 4:**
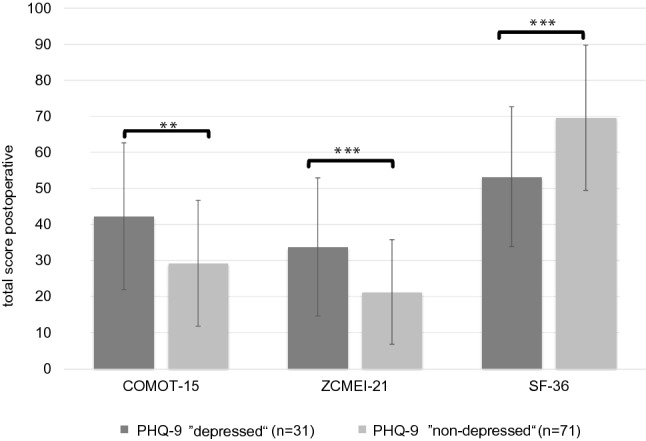
Postoperative assessment of COMOT-15, ZCMEI-21 and SF-36: comparison of preoperative depressed versus non-depressed patients on the categorical PHQ-9; PHQ-9 nine-item depression scale of the patient health questionnaire; ***p ≤ 0.001 depressed versus non-depressed; ***p ≤ 0.01 depressed versus non-depressed

**Fig. 5:**
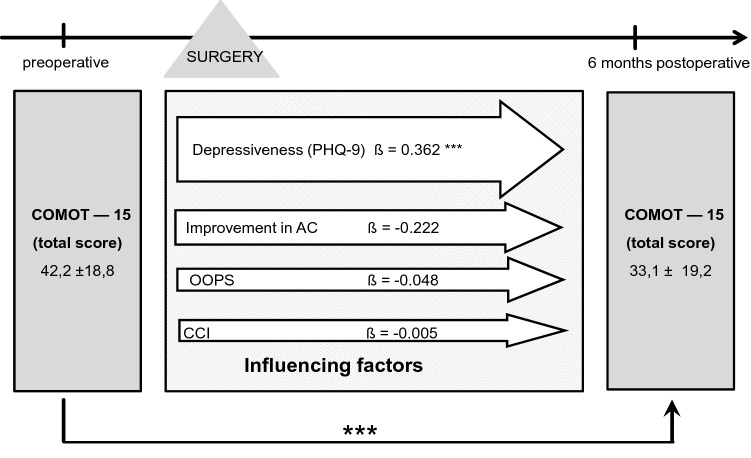
Multiple regression analysis of influencing factors on postoperative COMOT-15; *CCI* Charlson comorbidity index; *OOPS* ossiculoplasty outcome staging index; AC, air conduction threshold; Data shown are mean ± SD; *β* regression coefficient; ***p ≤ 0.001

**Fig. 6:**
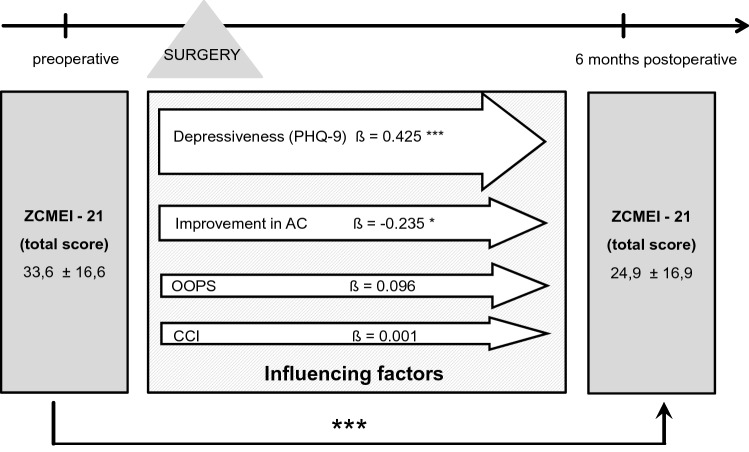
Multiple regression analysis of influencing factors on postoperative ZCMEI-21; *CCI*, Charlson comorbidity index; *OOPS* ossiculoplasty outcome staging index; *AC* air conduction threshold; Data shown are mean ± SD; *β* regression coefficient; ***p ≤ 0.001; *p ≤ 0.05

- *HRQOL measurement*
  Middle ear surgery resulted in significantly better total COMOT-15 and ZCMEI-21 scores postoperatively (Fig. 1). However, when comparing pre- and postoperative results, no significant change in the total SF-36 score was found (Fig. 2).
- *Impact of postoperative hearing level on postoperative HRQOL*
  The mean preoperative AC was 48.9 ± 22.5 dB, and this changed to 47.1 ± 23.8 dB postoperatively (*p* > 0.05). Eleven patients (10.8%) had a postoperative AC of 20 dB or lower, 34.3% (35) patients an AC of 21–40 dB, 26.5% (27) an AC of 41–60 dB, and 28.4% (29) an AC of > 60 dB. No significant difference was found between the pre- and postoperative bone conduction thresholds (mean difference − 2.9 ± 8.8 dB, *p* > 0.05).
  Correlation analyses were performed between both the postoperative COMOT-15 and ZCMEI-21 scores and postoperative AC. A moderate association between the postoperative audiometric data and the postoperative overall scores of COMOT-15 and ZCMEI-21 was observed (Fig. 3).
- *Impact of depressiveness on HRQOL*
  Generally, depressiveness measured by PHQ-9 scores did not change after middle ear surgery (mean difference 0.9, *p* > 0.05). Of the participants, 31 (30.1%) with COM had a PHQ-9 score of 5 or greater, indicating elevated depressive symptoms preoperatively. The mean postoperative COMOT-15 and ZCMEI-21 scores were significantly higher in patients with preoperative depressive symptoms than in patients without depressive symptoms (*p* < 0.001, Fig. 4). Patients with preoperative depressive symptoms reported significantly lower SF-36 scores, indicating worse general HRQOL in the postoperative setting (Fig. 4).
- *Multivariate analysis*
  Multivariate analyses for postoperative disease-specific HRQOL were performed with explanatory variables including middle ear status, comorbidity, depressiveness, and hearing improvement. The results of the multiple regression analyses showed that, even when adjusting for the OOPS index, CCI, and improvement in AC, preoperative depressiveness remained significantly associated with higher COMOT-15 (Fig. 5) and ZCMEI-21 (Fig. 6) scores postoperatively. If the hearing improvement (change in AC) was replaced by the postoperative AC in this regression model, the effect was almost the same (data not shown).

## Discussion

Satisfactory surgical, functional, and HRQOL outcomes have been well documented after reconstructive middle ear surgery [3, 4, 8–11, 32, 33]. However, so far almost all studies have neglected the psychopathological predisposition of patients.

In Germany, the prevalence of current depressive symptoms is approximately 8.1% (women: 10.2%; men: 6.1%) [34]. In our survey of 102 patients with COM, 31 patients presented with clinically relevant depressive symptoms. Depression rates (indicated by PHQ responses) in COM patients are similar to those of chronic disease populations. Similar results were obtained in rhinological studies elucidating the prevalence of depressive disorders in patients with chronic rhinosinusitis [35].

Depression is associated with various chronic medical illnesses, including coronary heart disease and diabetes [36]. The bidirectional relationship between a chronic disease and comorbid depression hinders unequivocal assessment of causality. However, it has been proven that comorbid depression negatively influences treatment outcomes. Patients with depressive disorders were shown to have a higher risk of adverse outcomes, increased analgesic consumption, and increased use of the healthcare system [20]. In analyzing the post-interventional outcome in patients with chronic diseases, such as degenerative lumbar spine disease, previous studies indicated a strong interaction between preoperative impaired mental health status and patient-reported outcome [17, 18]. In otorhinolaryngology head and neck surgery (ORL HNS), the association between psychological comorbidities and post-treatment outcome has been minimally investigated. The coexistence of depressive disorders and head and neck cancer [37, 38], chronic rhinosinusitis [39, 40], allergic disorders [41], tinnitus [42], and hearing disorders [12–14] is well described. However, the impact of psychological burden on PROMs after treatment was assessed in only a few studies. In patients with chronic rhinosinusitis, the preoperative disease burden was higher in patients with depressive disorders. Additionally, postoperative improvement in disease-specific HRQOL after endoscopic sinus surgery was significantly lower in patients with depressive symptoms [43]. In patients undergoing cochlear implantation, HRQOL was shown to be influenced by depressive symptoms, anxiety, and the perception of stress [44, 45].

In this study, we observed a general significant improvement in disease-specific HRQOL after middle ear surgery. Postoperative HRQOL correlated moderately with the postoperative AC, which is consistent with former HRQOL analyses [3, 8, 11]. However, the results of our multiple regression models stress the importance of preoperative depressiveness as the main predictor of postoperative HRQOL after middle ear surgery. Furthermore, otological studies mainly addressed pathology and surgery-related factors that may influence the HRQOL outcome. Univariate analyses of potential influencing factors, such as the presence of cholesteatoma, surgical techniques, revision surgery, mastoid obliteration, and bilateral disease, showed heterogeneous results [3, 10, 11, 46, 47]. Considering these results, patients with normal postoperative clinical observations (intact tympanic membrane, dry ear, and sufficient improvement in AC) and a low disease-specific HRQOL should be screened for concomitant depressive symptoms and, if necessary, offered a specific supportive therapy.

Previously, minimal attention has been devoted to managing depressive disorders in patients with COM and investigating how the management of depressive disorders may affect COM-specific HRQOL after surgery. However, our results suggest that modulation of mood disorders may open new doors to improve HRQOL in COM patients. It may be important to address comorbid depressive disorders as a component of comprehensive management of patients with increased depressiveness and COM. In the presence of manifest psychological disorder, appropriate therapeutic cooperation should, therefore, be discussed even before surgery. Our results support the need for interventional studies to test and confirm this hypothesis.

In ORL HNS, the feasibility and benefit of accompanying psychotherapy was mainly analyzed in cancer treatments [48]. Psychological intervention enables psychological counseling and support, increases psychological perseverance, and reduces negative psychological and adverse social reactions [49, 50]. To analyze the effect of psychological intervention on postoperative outcomes, high-quality, prospective trials are required with baseline psychological evaluation, standardized interventions, and integrated reporting of quality of life, psychological, and physiological outcome measures [46]. Reconstructive orthopedic studies, which analyzed the effectiveness of psychological intervention in hip or knee arthroplasty, showed heterogeneous results in terms of functional outcome and pain control [51]. However, the quality of prior studies has not been ideal due to the lack of long-term outcome and the ignored demand effect. Presumably, patients with increased depressiveness may benefit more from psychological intervention than those without an impaired mental health status [51]. So far, no similar investigations have been published for non-oncological ORL HNS.

General HRQOL remained stable over the observation period and did not improve significantly after middle ear surgery in our study. These results are consistent with those of other studies, which analyzed general HRQOL after ear surgery [3, 4]. Specific symptoms that may affect everyday life are insufficiently represented by general HRQOL measurement instruments. However, general HRQOL instruments are necessary to measure the impact of specific diseases on general health and allow comparisons that determine the impact of different diseases on general HRQOL [3].

While the data were prospectively collected, only a few patient demographics and comorbidities were included in our data. We may have failed to capture other factors that are important in patients’ perception of COM and its treatment. Our psychosocial assessment mainly focused on depressive symptoms. No other psychiatric or psychosomatic disturbances were analyzed. Particularly, the impact of anxiety, personality, and somatization should be investigated in more detail in further investigations, as studies from other subject areas demonstrated possible effects on PROMs [20, 23]. Furthermore, we must acknowledge a selection bias, as only symptomatic patients underwent middle ear surgery and were included. Asymptomatic patients are not usually sent to our tertiary referral center. Therefore, it was impossible to provide an adequate control group. Additionally, it is noteworthy that the study did not consider previous diagnoses of a depressive disorder or past medical treatment or psychotherapy. The extent of depressiveness was determined by a self-report inventory and was not supplemented by a psychological examination of a psychiatrist or clinical psychologist. Diagnosis of depression has traditionally been based on clinical criteria, including patients’ symptoms and history. However, to standardize data and interpretations, various interview-based instruments for depression screening were developed and used in both clinical and research practice. For the diagnosis of depressive disorder, the PHQ-9 showed excellent criterion validity in medical patients, with a sensitivity of 95% and a specificity of 86% [28]. It takes approximately 5 min to complete the questionnaire. Evaluation of the PHQ-9 takes approximately 2 min. Diagnostic validity and easy application make it a practicable tool even in busy clinical practice settings.

In the perioperative setting, the evaluation of patient-related factors and how they influence the patient’s surgical candidacy is ever more crucial in value-based care models. Depressiveness is a pertinent comorbidity that should be included in that evaluation. Consequently, we recommend routine preoperative screening of COM patients for depressiveness to initiate differentiated psychological supportive care.

## Conclusion

Depressive disorders occur with increased prevalence in patients with COM. The presence of comorbid depressiveness with COM leads to poorer HRQOL outcomes after reconstructive middle ear surgery. Therefore, improvements are required in screening, diagnosis, and treatment for patients with comorbid depressive disorders and COM. Simultaneously, if no improvement is observed in the measured HRQOL, an unknown depressive symptomatology must be considered if a clear clinical improvement is observed in the disease symptoms.

## Data Availability

Not applicable.
